# Analisys of high frequency auditory thresholds in individuals aged between 18 and 29 years with no otological complaints

**DOI:** 10.1016/S1808-8694(15)31069-7

**Published:** 2015-10-22

**Authors:** Leonardo Conrado Barbosa de Sá, Marco Antonio de Melo Tavares de Lima, Shiro Tomita, Silvana Maria Monte Coelho Frota, Gisele de Aquino Santos, Tatiana Rodrigues Garcia

**Affiliations:** 1 Medical residency in Otorhinolaryngology at the Rio de Janeiro State University, Master's degree student in the Medical Graduate Program - General Surgery, focused on Otorhinolaryngology.; 2 Doctor in Medicine - Otorhinolaryngology, Adjunct Professor of Otorhinolaryngology of the Rio de Janeiro Federal University Medical School.; 3 Doctor in Medicine - Otorhinolaryngology, Professor Titular de Otorrinolaringologia da Rio de Janeiro Federal University Medical School.; 4 Doctor in Speech Therapy, Adjunct Professor of the Speech Therapy Course at the Rio de Janeiro Federal University Medical School.; 5 Student of the speech therapy course at the Rio de Janeiro Federal University Medical School.; 6 Student of the speech therapy course at the Rio de Janeiro Federal University Medical School.; 7 Otorhinolaryngology Unit - University Hospital Clementino Fraga Filho - Rio de Janeiro Federal University.

**Keywords:** audiometry, hearing, auditory threshold, hearing loss - high-frequency, diagnostic techniques and procedures

## Summary

**R**ecent studies analyzing audibility thresholds at frequencies over 8 KHz have brought new perspectives on the investigation of auditory damage. These studies, however, have not yet reached a consensus on normal standards for auditory thresholds at these frequencies. **Aim:** To analyze the results of high frequency auditory thresholds in individuals aged between 18 and 29 years with no otological complaints. **Type of Study:** A prospective, cross-sectional study. **Methods:** 60 conventional audiometries were done and 51 of these exams were within normal limits in individuals aged 18 to 29 years. These selected individuals underwent high-frequency audiometry using the AMPLAID 460 device and Sennheiser HD 520 II earphones, and thresholds were obtained in dB HL Results: There was no significant difference in auditory thresholds between males and females. High-frequency auditory thresholds were obtained for individuals with no otological complaint, aged between 18 and 29 years. **Conclusion:** It has been suggested that such data could be used as a normal reference for further studies with similar standard equipment, to analyze auditory alterations presented in young individuals.

## INTRODUCTION

High frequency audiometry was introduced into clinical practice in the beginning of the 1960s,1 although initial research had been done in the first half of the 19th century. Until the 1960s, there was little interest in assessing high frequency auditory thresholds, given the conclusions of studies at the time that early auditory damage could be detected by evaluating auditory thresholds up to 8 KHz.^2^,^3^

Dadson and King4 published a study on standardization of audiometers in which 18% of subjects aged between 18 and 25 years did not respond to sound stimuli at 15 KHz.

Rudmose^5^ developed a Békésy-type high frequency audiometer for clinical use. This audiometer used Brüel & Kjaer microphone as a transducer inserted into the patient's external auditory canal using a plastic conical mold. In 1961 a study was published on auditory thresholds up to 18 KHz in high-school teenagers. Although the sample was small (12 subjects), the study pioneered this field.^5^

Fletcher further developed and calibrated this model,6 and wrote that although there was a consensus that the human ear responded to high frequency sound stimuli, little was known about the auditory capability in man. Furthermore, wrote Fletcher, there was no standardization of auditory values for frequencies over 8000 Hz; there were technical obstacles to assess ultra-high frequency thresholds, such as the lack of equipment capable of generating adequate pressure sounds at high frequencies, earphone models, calibration and standardization. The author tested the reliability of auditory thresholds using two devices and different tests, and correlated the results at common frequencies between both audiometers. The study included ^15^ North American soldiers from Fort Knox, Kentucky, aged between 18 and 25 years. These subjects underwent auditory threshold investigation done with two audiometers, a Békésy-type ARJ-4 HF, that examined frequencies between 4 and 18 KHz, developed by Doctor Wayne Rudmose and manufactured by Tracor, Inc., and a conventional Rudmose ARJ-5 audiometer, used for comparison. Provisory audiometric zero values were established for high-school students, and NPS thresholds were not obtained. Retest reliability for the Rudmose audiometer was checked at 4, 6, 8, 9, 10, 11, 12, 13, 14, 15, 16, and 18 KHz. Each soldier was tested three times for conventional and high frequency audiometry. Fletcher concluded that high frequency audiometry was reliable, but that caution was needed when comparing results obtained from different techniques and equipment.

Fausti et al.^7^ reported the development of a new audiometer, and described the stimulus generation model, the transducer, calibration, and electroacoustic analysis. A case study was presented, and the main difficulties of high frequency audiometry at the time were described, such as the lack of maximum power to assess hearing loss adequately, adaptation problems, earphone quality and positioning, signal fidelity after amplification, and audiometer calibration. Follow-up of the study with a larger sample led to the conclusion that conventional audiometry could erroneously provide a normal result due to the limited number of frequencies. High frequency audiometry could amplify, confirm and/or refute clinical impressions provided by audiometry up to 8 KHz, allowing early diagnosis, the description, and the differentiation of noise-induced auditory loss.^8^

Stevens et al.^9^ wrote that continuous sound waves in the external auditory canal were unable to provide a basis for assessing high frequency auditory thresholds due to uncertainties in the specification of acoustic stimuli. The authors suggested a calibration procedure to estimate the sound pressure at the inner end of the external acoustic canal.

In 1985 a historical review of high frequency audiometry compared the results obtained through free-field high frequency audiometry and results from conventional earphones.10 AAF (8 to 18 KHz) was done in a group of 10 males and 10 females aged between 20 and 29 years, with conventional auditory thresholds below 15dB NA. The result was that although all subjects responded to all of the tested frequencies, there was an abrupt threshold increase over 14 KHz in free-field audiometry, while testing with earphones showed a progressive increase over 12 KHz. The authors concluded that methods using earphones are more sensitive and easier to use.

Schechter et al.^11^ selected 157 subjects, 94 male and 63 female, aged between 6 and 30 years with normal auditory sensitivity (<15 dB re:ANSI 1969), for AAF using the same audiometer and a KOSS HV/IA earphone employed in Fausti et al's paper.^7^ According to these authors, AAF normality thresholds were not reliable, although they offered some standardization models. Results showed that there was a response to all frequencies up to 16 KHz in all age groups, and that the auditory capacity were decreased at frequencies over 10 KHz with progression of age. There was a 100% response rate at 20 KHz in the 6 to 10 year age group, and a 44% response rate in the older group (26-30 years). Auditory quality was also more variable in older subjects, which led the authors to question the causes of high frequency auditory quality loss in young people as age progressed. Genetic and pre and post natal causes were suggested. In the three main age groups of the study (6-10 years, 11-15 years, and 16-20 years), the authors found an average 2.5 times increase in auditory thresholds at 12 to 18 kHz. The authors concluded that their data appeared to confirm a gradual loss of sensitivity at high frequencies as age progressed from infancy to adolescence and to the first years of adult life. The authors made two suggestions: that studies on an older population would be needed to prove a progressive loss of thresholds with age and variation within a group according to age, and that subjects that for any reason used ototoxic drugs should have a baseline AAF and that a 15 to 20 KHz variation between tests should be defined as resulting from the treatment, since normal reference values had not been yet established.

Green et al.^12^ investigated auditory thresholds between 8 and 20 KHz in 37 young adults (18-26 years) with no otological complaints, using a new high frequency audiometer. All of the subjects had 15 dB NA or less at all frequencies up to 8 KHz, and a normal immitance test. The authors used an insert earphone calibrated for each tested ear, and retested the subjects to estimate the mean high frequency auditory threshold in subjects with normal hearing. A microphone was implanted in the part that was inserted in the external acoustic canal to measure the response when the audiometry device generated an impulse. The difference between the sound pressure level emitted by the device and that perceived in the middle of the external auditory canal was calculated for this response. This difference increased gradually from 2 to 12 dB as the frequency increased. The result was that the mean threshold was 23 dB at 8 KHz, 30 dB at 12 KHz, and dB 87 at 18 KHz. The authors reported a 15 dB difference between emitted sound pressures and those in the inner half of the external auditory canal. They concluded that comparisons between studies using insert earphones and those using conventional earphones were limited, and that the anatomy of the external auditory canal could alter AFF evaluation.

Frank and Dreisbach^13^ conducted a study on the reproducibility of high frequency auditory thresholds in the same subject after four tests in series using a Beltone 2000 audiometer. Fifty volunteers were included, 25 male and 25 female with a mean age of 22.6 years and auditory thresholds within normal limits (≤15 dB NA; ANSI S3.6-1989) at conventional frequencies (0.25-8 KHz) and normal immitance tests. Auditory thresholds were obtained at 10, 12, 14, 16, and 18 KHz for each subject in four test sessions with at least a one week and not more than a two week interval using earphones fitted by the examiner. Differences between auditory thresholds for each of six possible comparisons between tests showed no significant difference (p > 0.05), reaching not more than 10 dB in 94% of the ears. According to these authors, these indices confirm the reproducibility of intra-subject auditory thresholds in a test sequence, and increase the importance and reliability of AAF for monitoring patients exposed to ototoxic drugs.

Tang and Letowski14 approached the problem of auditory threshold variability at high frequencies between similar persons and the difficulty of audiometric calibration at high frequencies due to variations in sound pressure levels for each external auditory canal. The study aimed to establish whether the use of insert earphones could reduce inter-subject threshold variability allowing future high frequency guidelines as those available for conventional audiometry. Ten young adults were selected, 5 male and 5 female, aged between 18 and 25 years, with no otological findings, and with thresholds up to 8 KHz below 15 dB NA. The authors used a high frequency Beltone 2000 audiometer with Sennheiser HD-250 earphones and Etymotic ER-1 insert earphones placed by the examining physician. Frequencies of 10, 12, 14 and 16 KHz were tested in two sessions. The authors found no significant difference between thresholds for the right and left ears or for the two test series. A further result was that insert earphones could slightly reduce response variability between subjects, which could facilitate normatization of auditory thresholds for frequencies over 8 KHz.

Burén et al.^15^ did pure tone audiometry at 250 to 20000 Hz in three groups with mean ages of 10.1, 14.6, and 18.8 years, totaling 335 subjects. They used an Interacoustics AS 10HF audiometer for high frequencies and a Koss/1A earphone. At ultra-high frequencies they found a systematic auditory threshold increase over 14 KHz in the 14-year and 18-year age groups, compared to the 10-year age group, which is similar to Schechter et al's11 study. A few studies did not show a variation in auditory thresholds between ages 10 and 20 years.16,17 The authors concluded that ultra-high frequency hearing begins to deteriorate at an age below 14 years.

Kenna et al.^18^ reported that one of the limiting factors for including audiometry over 9 KHz in the clinical routine is the lack of additional normative studies to establish hearing at these frequencies. The authors listed the factors that interfere with testing over 4 KHz, including complex interactions between stimulus wavelength and the dimensions of the external auditory canal, difficulties in calibrating the equipment, and the signal-to-noise ratio. The authors did audiometric tests on 60 children aged between 5 and 18 years at 0.25 to 20 KHz and reported decreased thresholds over 14 KHz. According to their results, normal high frequency hearing could not yet be established, but audiometry over 8 KHz was sufficient to monitor a subject before and after exposure to ototoxic drugs.

Fouquet^19^ did AAF on 60 subjects equally divided between genders, aged between 18 and 30 years, using an Interacoustics AS10 HF audiometer and Koss HV/PRO earphones, presenting results in NPS. The authors noted a statistically significant difference between right and left ear thresholds according to gender, age group and frequency in only 3 of 40 statistical analyses. In their study there was a relatively linear audiometric curve at 9 to 12 KHz, and an abrupt threshold decrease over 15 KHz in the 18 to 24 year age group, and over 13 KHz in the older age group. The authors reported a decreased hearing acuity for ultra-high frequencies as age progressed.

Azevedo and Iorio3 assessed 52 subjects, 32 males and 20 females aged between 12 and 15 years, to establish high frequency auditory thresholds. The authors used an Interacoustics AS10 HF audiometer and Koss HV-1A earphones; thresholds were given in NPS. Results showed no statistically significant difference between auditory thresholds for both ears except at 1 KHz. There was no gender difference. The authors noted that high frequency auditory threshold means and medians were stable at 13 KHz, and that there was a gradual threshold increase above 14 KHz. This study concluded that there was a statistically significant difference between auditory thresholds at 9 to 18 KHz in both ears.

Sahyeb et al.^20^ reported that there were many problems with high frequency auditory threshold assessment methods, such as absence of a consensus on the importance of sound, poor calibration standards, audiometer and earphone limitations, variation between investigation methodologies, and mostly a lack of consensus between results. The authors studied 50 subjects (24 male and 26 female) aged between 18 and 30 years, which were audiologically within normal limits according to the clinical exam and conventional audiometry (ANSI S.3,6).21 High frequencies were assessed using a SIEMENS SD50 audiometer and SENNHEISER HDA200 earphones, NA-corrected according to certificate number 1.51-9493/92 and 14738/93. Frequencies of 9, 10, 11.2, 12.5, 14, and 16 KHz were investigated. Two examiners were responsible for doing the tests, to analyze intra-subject variation. The examiners placed the earphones for the first test at the abovementioned frequencies, and the examinee placed the earphones for testing at 9 and 16 KHz only to analyze the variability due to earphone positioning reported in literature. Research subjects were briefly trained to improved high frequency pure tone perception. Four high frequency audiograms were done for each subject, including four threshold studies at 10 and 14 KHz and eight threshold studies at 9 and 16 KHz. The investigators tried to reduce confounding variables by randomly presenting the various frequencies and randomly choosing the initial test ear. Results showed no significant difference between auditory thresholds in males and females or between ears. The authors reported improved auditory sensitivity due to the increased frequency, with auditory thresholds around 3 dB NA at 9 KHz and - 4dB NA at 16 KHz. The authors also underlined that this auditory quality improvement in young subjects probably was due to calibrating the device in NA, where most of the previous studies assessed thresholds using dB NPS, demonstrating decreased auditory acuity with increased frequencies. According to the investigators, this feature of previous studies reflects the physiology of the human inner ear that requires higher sound power to detect high frequency tones. Another finding was that mean thresholds at those frequencies were never over + 5 and - 5 dB NA. There was no significant variability in this study due to earphone placement by the examiners, or the examinees, or by different examiners. There was, however, a threshold difference in the same subject when tests were done in different days. The authors concluded that improvements obtained in the second day could be due to learning. There was also increased inter-subject variability due to increased frequencies, seen most clearly above 12 KHz. The authors concluded that prior training before the test, particularly for frequencies over 12 KHz, could increase test reliability, and that high frequency auditory monitoring should be compared individually, and not between subjects, due to variability.

Although high frequency devices were available for these studies, there was no consensus in results or in the assessment of the importance of these sounds. These papers highlight the lack of fidelity to calibration standards, audiometer and earphone limitations, the complex interactions between wavelength and the dimensions of the external auditory canal, and the significant variation between tests. These concerns demonstrate the paucity of knowledge about normality and disease.^3^ Furthermore, most papers present results in sound pressure levels.

Further consistent and serial studies are needed to investigate high frequency auditory threshold standards in subjects with no auditory complaints in various age groups. These studies could further knowledge about normalcy, facilitating early detection of auditory deficiency, particularly sensorineural hearing loss, which usually begins at higher frequencies.

This paper aims to analyze the results of high frequency auditory threshold testing in subjects aged between 18 and 29 years with no otological complaints.

## PATIENTS AND METHOD

This is a prospective, cross-sectional study. The research protocol was analyzed and approved by the Research Ethics Committee, fulfilling all of the requirements for clinical investigation in human beings.

Subjects were selected from volunteers that had sought the Otorhinolaryngology unit between June and September 2005, through a poster in the hospital facilities asking for men and women aged between 18 and 29 years with no otological complaints.

Subjects were informed about the aims of the study and the required procedures. All subjects decided to participate in the study and signed a free informed consent form. They were also informed that there was no cost involved in participation and that they were free to leave at any stage.

Exclusion criteria were subjects with a history of chronic otological disease, otological surgery, acoustic trauma, an altered auditory threshold in a previous test, a family history of hereditary otological disease that led to hearing loss, a profession involving frequent exposure to noise, patients that were not interviewed or that did not undertake audiometric tests, or that had a conventional audiometry test with a threshold over 25 dB NA at any frequency.

We carried out 60 conventional audiometric tests, of which 51 tests were within normal limits. These 51 subjects (32 female and 19 male) became the study sample. Volunteers were interviewed using a standard questionnaire applied by a single otorhinolaryngologist.

The data collection tool investigated social and demographic variables and potential associated factors causing variations in auditory thresholds at different age groups.

The next stage involved an otomicroscopic exam done by the same otorhinolaryngologist to exclude conditions that might interfere with auditory threshold testing. At this point three subjects were excluded due to clinical findings, such as tympanic membrane perforation or tympanosclerosis, and were referred to the Otorhinolaryngology unit.

Selected volunteers underwent conventional pure tone audiometry using an Amplifon AMPLAID 460 audiometer and a Telephonics 296 D 100-1 conventional earphone. Four speech therapists monitored by an otorhinolaryngologist applied the tests in an appropriately soundproofed audiometry booth.

Subjects were placed in the soundproofed booth and positioned on a chair facing away from the examiner. The examiner placed the earphones over the subject's ears and closed the booth.

There were 21 subjects that presented auditory thresholds below or equal to 25 dB NA between 250 and 8000 Hz. These subjects undertook high frequency audiometry. Six subjects were excluded at this stage.

The same equipment was used for the second test in the same booth, with Sennheiser HD 520 II earphones specific for high frequencies; thresholds were given in dB NA. Auditory thresholds were investigated between 9 and 18 KHz, at 1000 Hz intervals (Picture 3).

Conventional and high frequency pure tone audiometry were done using the descending technique.22

Statistical analysis was as follows:

- The Mann-Whitney test was used to compare thresholds between males and females;
- Wilcoxon's signed rank test was applied to analyze the threshold variation between right and left ears;
- Friedman's Analysis of Variance was done to analyze threshold variation at different frequencies. The multiple comparison test based on Friedman's statistics was applied to identify which frequencies differed from each other.^23^
- Non-parametric tests were used, as the variable threshold did not have a normal distribution (Gauss distribution) due to data dispersion and a lack of distribution symmetry. The significance level was 5%.

## RESULTS

We assessed the auditory threshold variation between males and females to find whether auditory thresholds were statistically different between genders. Tables 1 and 2 show the mean, the standard deviation (SD), the median, and the minimum and maximum threshold values according to sex, and the corresponding descriptive level (p-value) for right and left ears. Mann-Whitney's test was used for statistical analysis.

**Table 1:** Statistical analysis of right ear auditory thresholds according to gender.

Code	Variable	Sex	n	Mean	S.D	Median	Minimum	Maximum	p value
X28	8 RE	male	19	6,1	6,8	5	-5	20	0,51
		fem	32	4,7	6,2	5	-5	20	
X30	9 RE	male	19	5,0	5,5	5	-5	15	0,57
		fem	32	3,8	6,0	5	-10	15	
X32	10 RE	male	19	4,5	8,5	5	-10	25	0,79
		fem	32	4,2	7,6	5	-10	20	
X34	11 RE	male	19	6,1	9,4	5	-10	30	0,37
		fem	32	3,4	7,2	5	-10	20	
X36	12 RE	male	19	4,5	13,5	0	-10	40	0,58
		fem	32	1,1	7,0	0	-10	15	
X38	13 RE	male	19	6,3	17,5	0	-10	60	0,42
		fem	32	0,0	7,1	0	-10	15	
X40	14 RE	male	19	-1,3	12,9	-10	-10	35	0,68
		fem	32	-3,0	10,3	-10	-10	25	
X42	15 RE	male	19	0,8	14,4	-5	-10	40	0,14
		fem	32	-3,0	12,9	-10	-10	30	
X44	16 RE	male	19	6,3	18,1	0	-10	40	0,69
		fem	32	4,2	15,6	0	-10	40	
X46	17 RE	male	19	10,5	17,2	5	-10	35	0,68
		fem	32	8,1	14,8	2,5	-10	35	
X48	18 RE	male	19	19,7	14,6	25	-10	35	0,13
		fem	32	15,0	14,4	20	-10	35	

RE: right ear

S.D.: Standard Deviation

**Table 2:** Statistical analysis of left ear auditory thresholds according to gender.

Code	Variable	Sex	n	Mean	S.D	Median	Minimum	Maximum	p value
X29	8 LE	male	19	5,8	8,9	5	-10	25	0,69
		fem	32	5,5	8,0	5	-5	25	
X31	9 LE	male	19	1,8	7,1	0	-10	15	0,10
		fem	32	4,7	6,3	5	-10	15	
X33	10 LE	male	19	5,3	5,6	5	-5	15	0,97
		fem	32	5,8	9,3	5	-10	25	
X35	11 LE	male	19	6,6	8,2	5	-5	30	0,59
		fem	32	7,8	9,7	5	-5	45	
X37	12 LE	male	19	4,2	8,5	0	-10	25	0,68
		fem	32	4,7	8,0	5	-10	25	
X39	13 LE	male	19	2,9	8,4	0	-10	30	0,54
		fem	32	1,4	7,4	0	-10	25	
X41	14 LE	male	19	-5,0	9,3	-10	-10	25	0,56
		fem	32	-4,5	9,0	-10	-10	30	
X43	15 LE	male	19	-3,2	10,2	-10	-10	25	0,99
		fem	32	-3,6	10,4	-10	-10	40	
X45	16 LE	male	19	3,9	16,6	0	-10	40	0,97
		fem	32	2,2	13,4	0	-10	40	
X47	17 LE	male	19	5,3	18,0	-5	-10	45	0,39
		fem	32	6,7	13,8	5	-10	40	
X49	18 LE	male	19	12,6	16,4	15	-10	35	0,16
		fem	32	19,4	12,9	25	-10	35	

LE: left ear

S.D.: Standard Deviation

There was no significant difference in right ear auditory thresholds between males and females in the 18 to 29 age group.

There was no significant difference in left ear auditory thresholds between males and females in the 18 to 29 age group.

The variation of auditory thresholds between right and left ears was tested. Table 3 shows the mean, the standard error (SE), the median, the minimum and maximum absolute threshold variation between ears (right-left) and the corresponding descriptive level (p value) of the statistical test for the total sample. Wilcoxon's signed rank test was used for statistical analysis.

**Table 3:** Statistical analysis of threshold variation between ears (RE-LE).

Code	Variation	n	Mean	S.E	Median	Minimum	Maximum	p value
X60	Var 8000 Hz	51	-0,392	1,04	0	-15	15	0,63
X61	Var 9000 Hz	51	0,588	1,09	0	-15	25	0,67
X62	Var 10000 Hz	51	-1,275	1,16	0	-15	25	0,17
X63	Var 11000 Hz	51	-2,941	1,26	-5	-25	15	0,032
X64	Var 12000 Hz	51	-2,157	1,43	-5	-20	40	0,032
X65	Var 13000 Hz	51	0,392	1,65	0	-20	60	0,60
X66	Var 14000 Hz	51	2,353	1,62	0	-35	45	0,15
X67	Var 15000 Hz	51	1,863	1,98	0	-40	50	0,52
X68	Var 16000 Hz	51	2,157	2,00	0	-20	45	0,68
X69	Var 17000 Hz	51	2,843	1,98	0	-25	40	0,34
X70	Var 18000 Hz	51	-0,098	1,66	0	-25	25	0,87

S.E.: Standard Error

There was a significant threshold variation at 11000 Hz (p = 0.032) and 12000 Hz (p = 0.032) between the right and left ear. There was, however, no significant difference between ears at the remaining frequencies.

A descriptive analysis of high frequency auditory thresholds was given. The high frequency auditory tone threshold mean was lower or equal to 16.9 dB NA at all tested frequencies in subjects aged between 18 and 29 years, as shown on Table 4.

**Table 4:** Descriptive analysis of thresholds in the 18 to 29 year age group.

CREe	Threshold	n	Mean	S.D	Median	Minimum	Maximum
X28	8 RE	51	5,2	6,4	5	-5	20
X30	9 RE	51	4,2	5,8	5	-10	15
X32	10 RE	51	4,3	7,9	5	-10	25
X34	11 RE	51	4,4	8,1	5	-10	30
X36	12 RE	51	2,4	10,0	0	-10	40
X38	13 RE	51	2,4	12,3	0	-10	60
X40	14 RE	51	-2,4	11,2	-10	-10	35
X42	15 RE	51	-1,6	13,4	-10	-10	40
X44	16 RE	51	5,0	16,4	0	-10	40
X46	17 RE	51	9,0	15,6	5	-10	35
X48	18 RE	51	16,8	14,5	20	-10	35
X29	8 LE	51	5,6	8,2	5	-10	25
X31	9 LE	51	3,6	6,7	5	-10	15
X33	10 LE	51	5,6	8,0	5	-10	25
X35	11 LE	51	7,4	9,1	5	-5	45
X37	12 LE	51	4,5	8,1	5	-10	25
X39	13 LE	51	2,0	7,8	0	-10	30
X41	14 LE	51	-4,7	9,0	-10	-10	30
X43	15 LE	51	-3,4	10,2	-10	-10	40
X45	16 LE	51	2,8	14,5	0	-10	40
X47	17 LE	51	6,2	15,3	5	-10	45
X49	18 LE	51	16,9	14,5	20	-10	35

RE: right ear

LE: left ear

S.D.: Standard Deviation

We then used Friedman's analysis of variance to assess auditory threshold variation at all frequencies. Such analysis investigated significant variation (increased or decreased) at all frequencies. The multiple comparison test, based on Friedman's statistics, was used to reveal frequency differences.

Table 5 shows the result of Friedman's analysis of variance and the statistically different frequencies according to the multiple comparison test at significance 5%. The significance level was adjusted to 0.5% to control the Type I error (error a) that implies erroneously finding significant differences when subgroups are compared. Table 5 shows the significantly difference frequency pairs separately for the right ear (lower matrix) and the left ear (upper matrix). Friedman's analysis of variance revealed that there was a highly significant variation at all frequencies (p = 0.0001).

**Table 5:** Multiple comparison test between frequencies (KHz).

Frequencies (Hz)


X: significant at 0.5%

lower matrix: right ear

upper matrix: left ear

The longitudinal behavior of frequencies between right and left ears was very similar. There were differences, however, that explain not merging right and left ear data.

**Chart 1:**
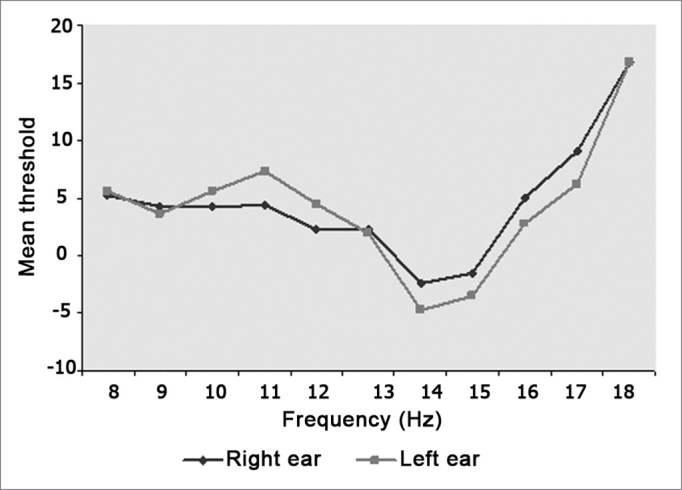
shows mean levels of right and left ear auditory thresholds.

## DISCUSSION

Conventional audiometry does not consistently assess the response capability of the base of the cochlea, a frequent site for hereditary and acquired conditions. This inner ear segment is vulnerable due to earlier maturity, local cell differences, specific cochlear mechanisms for each stimulated frequency that activate basilar membrane activation mechanisms, proximity to the oval and round windows, the biochemical composition, and vascularization along the cochlear duct, which result in greater exposure to pressure and toxin fluctuation.

An adequate evaluation of this cochlear segment using high frequency auditory thresholds still requires standardization for the type of equipment (dB NPS or NA), calibration, and earphone positioning, among others, eventually for reliable comparisons between studies.^8^,^10^,^1^ ^1^,^12^,^16^,^17^,^20^,^24^, ^25^, ^26^, ^27^

According to the statistical test results of our study, we found that there were no significant gender differences between auditory thresholds. These results are similar to those reported by Green et al.,12 De Seta et al.,10 Okstad et al.,1 Azevedo and Iorio,3 and Sahyeb et al.20 who studied young subjects, and to most of the available studies made on other age groups. Some papers report significant differences on one side only at limited frequencies, such as those published by Martinho et al.27 and Fouquet19 that found improved audibility in women only in the right ear and at 10 and 16 KHz and 14 KHz. Northern et al.28 published a study that included 237 patients aged between 20 and 70 years, and found that men had more uniform hearing loss than women between the third and fourth decade of life; the authors do not inform the comparative intensity of this. Pedalini et al.29 reported a significant gender threshold difference in subjects aged between 21 and 30 years, and 41 and 50 years (p<0.005), with best responses in women; the paper does not explain if this finding applies to all frequencies or to a single frequency. Two other papers17,30 reported decreased auditory acuity in men, on average 4.4 dB NPS at all frequencies and in consecutive age groups. Literature does not provide us with a consensus as to the superior auditory quality of women compared to men, but it is relevant to note that the opposite has not been observed in any published paper.

Our results show no significant auditory threshold variation between right and left ears when evaluating the total sample of volunteers. Two frequencies, however, showed significantly superior thresholds for the right ear. As to the significant variation between thresholds for both ears, we can only speculate. Various authors13,20,25,29,31,32 found no statistically significant variation in the quality of thresholds between ears. Some authors16,27 noted improved auditory thresholds for the left ear. This impression might have been caused by the fact that tests were always started on the right ear, and the left year would have learnt from the first test. According to Schechter et al.,11 thresholds tend to be symmetrical between ears during the first years of life, and variation develops with age, reaching values between 5 to 15 dB NPS, depending on age and the frequency. In our opinion, these results may depend on genetic and environmental factors, including exposure to noise and ototoxic drugs, which might explain the variation we found.

Mean results of high frequency auditory pure tone thresholds were equal to or less than 16.9 dB NA at all frequencies tested in subjects aged between 18 and 29 years. Auditory sensitivity was stable up to 13 KHz, then presenting significant improvement at 14 and 15 KHz, with a mean threshold value of 3.6 db NA at 9 KHz and - 4.7 dB NA at 14 KHz for the left ear. Over 16 KHz auditory thresholds increased bilaterally until 18 KHz. We found a curve with a trend towards linearity up to 15 KHz, over which the curve ascends. These values are similar to those described by Sahyeb et al.20 who used an audiometer with an upper frequency limit of 16 KHz and who published results in dB NA. These authors found 3.54 as the mean threshold at 9 KHz and - 4.55 at 14 KHz. Our results diverge somewhat from those found by Pedalini et al.,29 who reported mean values of 10 dB NA at 10 KHZ and 0 dB NA at 14 KHz. There is one other paper that gives results as NA, but the mean is obtained by grouping results of subjects aged between 15 and 50 years, which precludes a comparison due to age dispersion.32

Other studies employed audiometers that give results as dB NPS. A comparison between the means of most of the studies was not possible; only a correlation of curve morphology was possible.

Some studies used devices that gave results in dB NPS according to ANSI 3.621 guidelines, and showed increased auditory thresholds as a function of increased frequency, as in our study; this was usually more intense over 14 KHz, which suggests loss of auditory sensitivity in young adults according to frequency.3,10,11,12,16,17,1 9,28-30 Azevedo and Iorio3 investigated a younger group aged between 12 and 15 years and found that auditory thresholds remained stable until 14 KHz, with progressively increased thresholds over this frequency, which was statistically significant. These results are similar to those published by Stelmachowicz et al.17 and Kenna et al.18 who assessed 50 subjects aged between 10 and 20 years and 56 subjects aged between 5 and 18 years, and found increased auditory thresholds over 14 KHz. Fouquet19 noted worsened auditory acuity in two groups aged 18 to 24 years and 25 to 30 years at 12 to 18 KHz in males, and at 18 KHZ in females. The authors found that there was earlier frequency loss below 16 KHz compared to our results. Burén et al.15 assessed 335 youths aged between 10 and 18 years and found that reduced thresholds started at age 14 years. Although Lipscomb et al.33 and Stelmachowicz et al.2 did not mention a specific frequency for the beginning of acuity loss, they noted that there is a clear loss with increased frequency, and that when frequency increased, the number of responding subjects decreases. In our study we found improved auditory acuity at 14 and 15 KHz that takes the shape of a trough in the curve for this age group before the significant threshold increase seen at higher frequencies. A similar curve may be seen in the papers published by Zislis and Fletcher16 and Northern et al.,28 who investigated subjects aged between 11 and 18 years and a subgroup aged between 20 and 29 years. In both studies there is a plateau between 14 and 15 KHz in the former, and between 13 and 14 KHz in the latter paper. De Seta et al.10 found a similar slight improvement in auditory thresholds at 14 KHz, shown on a chart but not commented. None of these authors made any attempt to explain this finding. We also found no reference in literature or in physiology texts that might explain this finding.

Our study confirms previously published papers. We agree with authors that believe that high frequency audiometry should not be used singly as a diagnostic method12,29 as normal standards have not yet been defined. When audiometry has been done before exposure to a harmful stimulus, however, the method may be used for monitoring purposes and for an early diagnosis of ototoxicity and injury due to high sound pressure levels, using the initial auditory threshold obtained before exposure for comparison. Use of high-frequency audiometry for comparison purposes in the same subject may provide warning of extensive cochlear injury that might affect the patient's quality of life. Use of an ototoxic drug, for instance, might be interrupted at this point. Wider clinical use, however, requires greater homogeneity of data between studies to establish a normal standard for high frequencies.

This paper provides auditory thresholds given in dB NA at high frequencies for subjects with no otological complaints aged between 18 and 29 years. These data may be used as a normal reference by future studies using similar equipment to assess auditory changes in young subjects.

## CONCLUSION

Based on our data pertaining to the auditory behavior at frequencies between 8 and 18 KHz in subjects aged 18 to 29 years and normal hearing, we conclude that:

1. Results show homogeneity between right and left ear auditory thresholds, with significant variation between right and left ears only at 11 and 12 KHz.
2. There was no significant difference between male and female auditory thresholds in the 18 to 29 year age group.
3. Mean values for high frequency auditory thresholds for the right ear in subjects aged between 18 and 29 years were: 5,2 dBNA at 8 KHz; 4,2 dBNA at 9 KHz; 4,3 dBNA at 10 KHz; 4,4 dBNA at 11 KHz; 2,4 dBNA at 12 KHz; 2,4 dBNA at 13 KHz; - 2,4 dBNA at 14 KHz; - 1,6 dBNA at 15 KHz; 5,0 dBNA at 16 KHz; 9,0 dBNA at 17 KHz, and 16,8 dBNA at 18 KHz.
4. Mean values for high frequency auditory thresholds for the left ear in subjects aged between 18 and 29 years were: 5,6 dBNA at 8 KHz; 3,6 dBNA at 9 KHz; 5,6 dBNA at 10 KHz; 7,4 dBNA at 11 KHz; 4,5 dBNA at 12 KHz; 2,0 dBNA at 13 KHz; - 4,7 dBNA at 14 KHz; - 3,4 dBNA at 15 KHz; 2,8 dBNA at 16 KHz; 6,2 dBNA at 17 KHz, and 16,9 dBNA at 18 KHz.
